# Resilience Assessment of Complex Urban Public Spaces

**DOI:** 10.3390/ijerph17020524

**Published:** 2020-01-14

**Authors:** Hui Xu, Yang Li, Lin Wang

**Affiliations:** 1 School of Economics and Management, Chongqing University of Posts and Telecommunications, Chongqing 400065, China; xuhui@cqupt.edu.cn (H.X.); s180701025@stu.cqupt.edu.cn (Y.L.); 2 School of Management Science and Real Estate, Chongqing University, Chongqing 400045, China

**Keywords:** resilience assessment, complex urban public spaces, disaster preparedness, vulnerability

## Abstract

Risk events frequently occur in “complex urban public spaces” (CUPSs) and cause serious economic losses and casualties. To reduce the risks and enhance the system resilience, this paper formulates a theoretical framework to assess the resilience of CUPSs. Resilience is defined as the ratio of preparedness to vulnerability, according to the implication of the concept. Three-level practical indicator systems were established for these two dimensions, respectively. Furthermore, a hybrid approach combining the Analytic Network Process (ANP) and the Decision-Making Trial and Evaluation Laboratory (DEMATEL) was adopted. The Chongqing West Railway Station (the Station (W)) and the Lianglukou Rail Transit Station (the Station (L)) were used for a case study. The results showed that the Chongqing West Railway Station was more resilient to risks than the Lianglukou Rail Transit Station. Therefore, the proposed theoretical framework could be applied in assessing the resilience level of CUPSs. Resilience improvement strategies can be formulated according to the assessment results. Furthermore, the practical indicators could also provide references for urban disaster management.

## 1. Introduction

Over the last couple of decades, some specific urban public spaces, such as complex rail transit stations, integrated railway transportation hubs, and airport terminals, have been constructed to intensively utilize land resources and greatly facilitate people’s lives. The obvious characteristics of these kinds of urban public spaces are multi-layer structures and crowded clusters. Additionally, various components, including functional facilities and connections, in multi-layer structures show non-linear and emerging features. Therefore, the spaces present complexity and can be recognized as complex systems, and are thus known as “complex urban public spaces” (CUPSs) [1]. CUPSs enable multidimensional functions and facilitate public lives. However, the characteristics of CUPSs (their complex structure, multiple facilitates, and crowded clusters) result in many key management points, which increase management difficulties and operation risks. The system is easily threatened by different kinds of disasters, including natural disasters, accidents, public health events, and social security events. Many risk events have occurred in CUPSs. For instance, on 1 March 2014, a terrorist violence incident occurred at Kunming Railway Station in Kunming city, China. Additionally, a firecracker burning accident happened at Shanghai Pudong International Airport on 12 June 2016. Risk events cause serious negative impacts on CUPSs and even the whole society. Therefore, the risks of CUPSs should be considered.

Many efforts have been devoted to measuring some aspects of disasters, including social vulnerability, hazard exposure, risk mitigation efforts, and disaster preparedness, etc. The concept of “resilience” provides new and comprehensive directions for urban disaster management research. In 1973, Holling first applied resilience to describe the capacities of ecological systems [2]. Later, the concept was extended to the field of urban and disaster research. In recent years, resilience has gradually been applied to the risk management of infrastructure and community [3,4,5]. Various risk events frequently occur at CUPSs due to their complex characteristics. There is an urgent need to determine the level of existing risks and understand the response ability of CUPSs. Resilience is commonly defined as the ability to resist, absorb, adapt, and recovery from disasters. The main purpose of resilience research is to strengthen a system’s capacity for disaster response and provide measures to minimize losses. Therefore, as important infrastructure in urban areas, resilience research on CUPSs presents rationality and necessity. Different CUPSs have different levels of resilience. The higher the resilience of the CUPSs, the higher the system’s ability to prevent and respond to risk events. Resilience assessment is increasingly seen as a key component in explaining the determinants of disaster resilience, which is conducive to disaster risk reduction and system resilience promotion. This paper aims to quantify the resilience of CUPSs by fully considering their influence factors. According to the implication of resilience, it is defined as the ratio of preparedness to vulnerability. A hybrid approach integrating the Analytic Network Process (ANP) and the Decision-Making Trial and Evaluation Laboratory (DEMATEL) has been adopted in the resilience assessment.

The remainder of this paper is organized as follows: Section 2 is the literature review; In Section 3, a theoretical framework for assessing the resilience of CUPSs is established; Section 4 introduces the assessment methods, including the Analytic Network Process (ANP) and Decision-Making Trial and Evaluation Laboratory (DEMATEL); In Section 5, the proposed resilience assessment methodology is applied to study cases; Section 6 discusses the assessment results and provides corresponding suggestions for policymaker; Section 7 is the conclusions, and future research directions are also presented.

## 2. Literature Review

### 2.1. Resilience

“Resilience” originates from the Latin word “resilio” and means “to bounce back” [6,7]. The development of resilience has experienced a long history of multiple interconnected meanings in art, literature, law, science, and engineering [6]. In 1973, Holling applied resilience to the field of ecosystems [2]. Since then, resilience has gradually been used in urban research to maintain systemic operations and reduce potential risks. The US Department of Homeland Security conceptualized resilience as the ability to resist, absorb, restore, or successfully adapt to adversity or conditional changes [8]. Three main capacities can be concluded from different definitions of resilience: absorbability, adaptability, and restorability. Resilience can be interpreted as the ability of a system to resist disruption by reducing the initial negative impacts (absorbability), adapting itself to disruption (adaptability), and recovering from disruption (restorability). Based on these three resilient capacities, this paper considers resilience from two aspects, including disaster preparedness and system vulnerability. Disaster preparedness refers to the preparation of activities and resources designed to improve system capacities in advance, which corresponds to absorbability, adaptability, and restorability. System vulnerability analysis presents the potential risks of random or intentional disruptions, corresponding to adaptability and restorability. Therefore, resilience is defined as the ratio of preparedness to vulnerability [9,10].

Many advanced techniques have been integrated into resilience research to better understand and manage risks in modern urban areas. Maps and Geographic Information Systems (GIS) are important techniques that can be used to map hazards, vulnerabilities, and risks. However, mapping urban resilience poses some challenges as there is no generally accepted method for this. In 2010, although Renschler et al. proposed that GIS plays a major role in assessing system resilience, there is no clear method on how to apply it in practice [11]. Cariolet et al. filled this gap and developed methodologies for mapping urban resilience to disaster [12]. This is an important step in promoting the wide-ranging application of advanced techniques in urban resilience research. Some international organizations have also taken actions in response to urban disasters. In 2005, the United Nations International Strategy for Disaster Reduction proposed the Hyogo Framework for Action 2005–2015 [13]. Subsequently, the Sendai Framework for Disaster Risk Reduction 2015–2030 was adopted to further achieve a substantial reduction of disaster risk and economic, physical, social, and cultural losses, etc. [14]. Moreover, the project for 100 global resilience cities was also proposed by the Rockefeller Foundation to strengthen the resilience of global cities. Disaster research is closely associated with the theoretical exploration and practical application of resilience. Zhang et al. considered 56 prefecture-level cities in China to explore the spatial distribution of urban resilience and its influencing factors of uncertainty disturbances [15]. Schoch-Spana et al. produced a self-assessment toolkit—the Composite of Post-Event Well-being rubric—to predict post-disaster community function and resilience [16]. Tiernan et al. identified three important emerging themes: the socialization of responsibility for resilience, concentration on utilizing public private partnership to continue risk management, and the exploration of adaptive resilience [17]. Besides, numerous studies have been conducted to understand the concepts of vulnerability and resilience. Vulnerability has been defined as the state of sensitivity for disasters linked to resilience [18]. Salas and Yepes conducted urban vulnerability analysis and flooding was considered as one of the driving forces to promote urban vulnerability analysis [19]. Szewranski et al. developed a social-environmental vulnerability mapping method to assess the resilience of specific social groups to flooding [20]. In this paper, vulnerability was identified from the system internal structure and the external environment. When combined with disaster preparedness, a resilience assessment system of CUPSs can be constructed.

### 2.2. Resilience Assessment Indicator System

Studies related to the resilience indicator model and framework of the resilience assessment are summarized in Table 1. The taxonomies of resilience assessment indicators could provide guidance for developing the CUPS indicator system.

### 2.3. Resilience Assessment Approach

Based on the implication of resilience, system functionality Q(t) was used to quantify resilience:

(1) $$R=\int_{t_{0}}^{t_{1}}\left[100-Q(t)\right]dt$$

where $t_{0}$ is the time that a risk event occurs and $t_{1}$ is the time when the system is completely repaired. Bruneau et al. [32] further developed a general tool to quantify resilience through functional metrics in a more detailed manner (Equation (2)):

(2) $$Q(t)=100-\left[L\cdot F\cdot \alpha_{R}\right]=1-\left[L(t_{0E})\cdot f_{rec}(t,t_{0E},T_{RE})\cdot \alpha_{R}\right]$$

where $L(L(t_{0E}))$ is the magnitude of the loss function; $F(f_{rec}(t,t_{0E},T_{RE}))$ represents the recovery function after the time of event occurrence $t_{0E}$, which is shaped according to available resources and allocated during the recovery period, $T_{RE}$; and $\alpha_{R}$ is the functionality recovery factor. Then, Cimellaro et al. [33] improved Bruneau’s method and defined resilience as the average of system functionality Q(t) throughout the life cycle (Equation (3)), where $T_{0}$ is the system control time.

(3) $$R=\int_{t_{0}}^{t_{0}+T_{0}}[Q(t)/T_{0}]\mathrm{dt}$$

Furthermore, the system resilience $R_{s}$ was proposed as the integration of the individual subsystem resilience $R_{i}$:

(4) $$R_{s}=g(R_{1},\ldots R_{i},\ldots ,R_{n})$$

where g() is a function that determines the combination of individual subsystem resilience values in a way that reflects their interdependence and connectivity. Henry et al. [34] upgraded the conceptual framework by proposing a time-dependent quantifiable metric based on Bruneau’s resilience definition, in which the system experiences three distinct states (original state, disrupted state, and recovery state) and two transitions (system disruption and system recovery). The proposed fundamental formula in agreement with the concept of resilience is displayed as Equation (5), where R(t) is the system resilience.

(5) R(t)=Recovery(t1)/Loss(t0)

It could be concluded that resilience can be quantified as the variation of the system function performance Q(t). The system function performance Q(t) was considered as a key parameter to assess resilience, with a focus on the system itself. Moreover, considering the characteristics of the CUPSs—their (1) high spatial integration, (2) super network hub, (3) large underground space, and (4) crowd cluster—the resilience assessment of CUPSs should concentrate on function enhancements, disaster prevention and response, and risk mitigation. It is commonly believed that economic losses and casualties are inevitably caused in CUPSs once a risk event occurs. Therefore, pre-disaster prevention and system vulnerability analysis should be considered to avoid these losses as much as possible. In this respect, these two dimensions were selected to assess the resilience of CUPSs, based on the implication of resilience. The resilience index was defined as the ratio of the preparedness index to vulnerability index [9,10]. This assessment approach was derived from the resilience definition proposed by the United Nations International Strategy for Disaster Reduction [35], which aimed to maximize the preparedness potential and minimize the vulnerability considered.

(6) Resilience Index(RI)=Preparedness Index(PI)Vulnerability Index(VI)

where RI is the resilience score. If “RI>1”, it demonstrates that the system is more resilient towards risks. If “RI=1”, it means that the system just has enough ability to overcome its vulnerability. If “RI<1”, it indicates that the system is less resilient towards risks.

## 3. Resilience Assessment Framework of CUPSs

**Figure 1:**
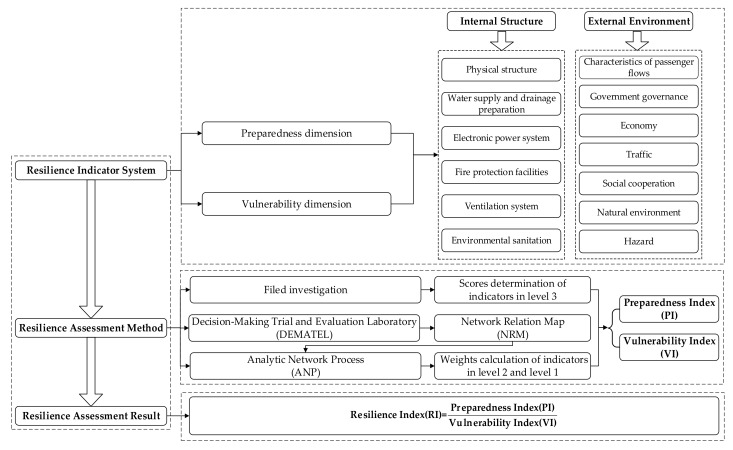
Resilience assessment framework.

To achieve a resilience assessment of CUPSs from preparedness and vulnerability dimensions, a theoretical framework with a hybrid approach that combined the Analytic Network Process (ANP) and the Decision-Making Trial and Evaluation Laboratory (DEMATEL) was formulated, as shown in Figure 1.

### 3.1. A Two-Dimension Practical Indicator System

#### 3.1.1. Preparedness Dimension

The European Commission Humanitarian Aid Office [36] stated that preparedness referred to measures taken by governments, organizations, communities, or individuals to guard against natural disasters or deliberate attacks and minimize their effect. Disaster preparedness is an increasingly important element in urban disaster management [37,38]. Discussions on the collaborative development of household, community, and organizational preparedness assessment strategies are theoretical innovations in disaster research. The preparedness of CUPSs is defined as emergency activities and resources that are prepared to enhance the system’s ability to respond to disasters and mitigate risks in effective manners.

#### 3.1.2. Vulnerability Dimension

Adger considered that vulnerability was the state of susceptibility to be harmed from the absence of adaptability and exposure to external stresses [18]. The vulnerability of transportation networks was described as a susceptibility to incidents, which can cause considerable reductions in network serviceability [39]. In this study, vulnerability is defined as the potential risk of CUPSs from random or intentional disruptions, which is mainly represented in the internal structure. The adaptability and restorability of the CUPSs become relatively weak after a disaster. Therefore, vulnerability assessment in an internal structure is particularly critical, and external environmental factors also play a vital role in vulnerability assessment.

### 3.2. Indicator Adaption to the CUPSs

The indicators extracted from the existing studies, in terms of community capacities, infrastructure, and resilience assessment, do not exactly match the characteristics of CUPSs. Therefore, modifications of the extracted indicators and the establishment of new indicators are necessary. Field investigations of typical CUPSs were conducted to collect information to determine indicators for every dimension. A top-down analysis approach was implemented to establish subcategories.

Based on the internal structure of the system, indicators in level 1 of the indicator systems of the “preparedness” dimension and “vulnerability” dimension can be summarized as the following six dimensions:

(1) *Physical structure*. CUPSs are characterized by multidimensional spaces and multi-layer structures, and several functions are integrated into the space. For instance, the traffic transfer hub integrates various transfer modes, including metro, bus, taxi, railway, etc. However, numerous random and intentional events are threatening CUPSs due to their characteristics of high spatial integration, large underground space, and crowded passengers. Therefore, the four main indicators in level 2 for analyzing preparedness and vulnerability can be identified as the *multi-layer structure*, *internal spatial layout*, *underground spatial layout*, and *multi-function*. Furthermore, the indicators in level 3 have been determined;
(2) *Water supply and drainage preparation*. This indicator is used to demonstrate the ability to prevent and respond to disasters such as fires and flooding. This dimension includes only one indicator in level 2, namely “*water supply and drainage facilities*”. The number of emergency water supply and drainage facilities is considered in level 3;
(3) *Electronic power system*. The *electronic power system* plays the backbone role in the normal operation of CUPSs. Indicators in the sub-level include *emergency power supply equipment* and the *power condition*;
(4) *Fire protection facilities*. *Fire-fighting equipment* is a general emergency tool for dealing with fire. Besides, the *automatic alarm system* can also remind passengers of emergency evacuation effectively and rapidly. The above two aspects are considered in the sub-level;
(5) *Ventilation system*. The *ventilation system* is associated with the air environment in CUPSs. In general, ventilation in three-dimensional space mainly depends on the air shaft and air conditioning system;
(6) Environmental sanitation. Environmental sanitation is related to the physical structure, ventilation system, characteristics of passenger flow, etc. Health protection measures should be considered as the main content, including post-disaster disease prevention and epidemic prevention. Disease and epidemic transmission can cause the expansion of accidents and even more serious losses. Post-disaster disease prevention is crucial and directly related to the recovery efficiency of the system. Accordingly, indicators in level 3 include “the number of cleaners” and “the number of sanitation and epidemic prevention personnel”.

In terms of the external environment, indicators in level 1 of the indicator systems in the “preparedness” dimension and “vulnerability” dimension are as follows:

(1) *Characteristics of passenger flows*. In CUPSs, crowd clusters exist at every layer, which is a vulnerable point to be struck by risk events. Therefore, passenger flow characteristics should be considered for emergency preparation and a rapid response. The sub-indicators in this dimension comprise *statistics of passenger flows*, *spatial and temporal distribution of passenger flows*, and *security protection for crowd clusters*;
(2) *Government governance*. In this dimension, the three sub-indicators are *multi-stakeholder cooperation*, *disaster emergency plan*, and *disaster management plan*. *Multi-stakeholder cooperation* represents the relationship between multi-government departments and enterprises, which is necessary for daily operations and the emergency response during a disaster. The *disaster emergency plan* aims to rapidly respond to risk events and reduce losses through pre-disaster planning and preparation. The *disaster management plan* includes security measures, such as disaster prevention propaganda, police security protection, and security facilities setting;
(3) *Economy*. Economic strength has some impacts on the emergency response speed during risk events. The level of economic development is relevant to the resilience of CUPSs. Therefore, the financial support and rescue material reserve offered by the government are considered. In addition, indicators reflecting regional economic development are also added;
(4) *Traffic*. *Traffic condition* is closely related to post-disaster emergency rescue. *Traffic accessibility* is an important indicator that can be demonstrated through *the number of connected roads* and *traffic operation*. High traffic accessibility facilitates rescuers and emergency vehicles;
(5) *Social cooperation*. *Social cooperation* includes *social preparedness* and *social service* from passengers, volunteers, and non-governmental organizations (NGOs). The mutual and close cooperation between professional rescuers and the public is conducive to disasters. The coverage of emergency vehicles and medical services is also considered;
(6) *Natural environment*. The *natural environment* is used to explain disaster preparedness. *The statistics of multi-category natural disaster* is considered to help further predict and prevent natural disasters;
(7) *Hazard*. CUPSs are exposed to the natural environment and subject to various disturbances. Hazards occurring in the CUPSs can be described in terms of *variety*, *characteristics*, and *severity*.

The indicator systems of “preparedness” and “vulnerability” are shown in Table 2 and Table 3, respectively. The indicator system of “preparedness” includes 12 indicators in level 1, 26 indicators in level 2, and 79 indicators in level 3, and the indicator system of “vulnerability” includes 10 indicators in level 1, 19 indicators in level 2, and 41 indicators in level 3.

## 4. Methods

### 4.1. Preparedness and Vulnerability Score Calculation Methods

Referring to the previously proposed mathematical method [9], the preparedness score is calculated as the weighted sum of the scores of all indicators in level 1 (PD), and PD is calculated as the weighted sum of the scores of the corresponding indicators in level 2 (PS). PS is calculated as the average score of the corresponding indicators in level 3 (PC), as shown in Equation (7):

(7) $$PI=\sum_{i=1}^{i=NP}w_{i}PD_{i}\;{\mathrm{PD}}_{i}=\sum_{j=1}^{j={\mathrm{MP}}_{i}}u_{\mathrm{ji}}PS_{\mathrm{ji}}\;{\mathrm{PS}}_{\mathrm{ij}}=\frac{\sum_{k=1}^{k=LP_{j}}PC_{ijk}}{LP_{j}}$$

where PI is the preparedness score of the system. $PD_{i}$, $PS_{ji}$, and $PC_{kji}$ are the scores of the i th indicator in level 1, the j th indicator in level 2, and the k th indicator in level 3, respectively. NP, $MP_{i}$, and $LP_{j}$ are the number of indicators in level 1, level 2, and level 3, respectively. $w_{i}$ and $u_{ji}$ represent the weights of the i th indicator in level 1 and the j th indicator in level 2.

The vulnerability score is calculated in the same way as the preparedness score, as shown in Equation (8):

(8) $$\mathrm{VI}=\sum_{i=1}^{i=\mathrm{NV}}y_{i}VD_{i}\;{\mathrm{VD}}_{i}=\sum_{j=1}^{j=MV_{i}}x_{ji}VS_{ji}\;{\mathrm{VS}}_{ij}=\frac{\sum_{k=1}^{k=LV_{j}}VC_{ijk}}{LV_{j}}$$

where VI is the vulnerability score of the system. $VD_{i}$, $VS_{ji}$, and $VC_{kji}$ are the score of the i th indicator in level 1, the j th indicator in level 2, and the k th indicator in level 3, respectively. NV, $MV_{i}$, and ${\mathrm{LV}}_{j}$ are the number of indicators in level 1, level 2, and level 3, respectively. $y_{i}$ and $x_{ji}$ represent the weights of the i th indicator in level 1 and the j th indicator in level 2.

In order to obtain the scores of the indicators, a field investigation is firstly applied to assess the indicators in level 3. Subsequently, the weights of indicators in level 1 and level 2 are determined by ANP and DEMATEL, as illustrated in the following section.

### 4.2. Indicator Weights Determination Methods

ANP and DEMATEL are used in this paper to determine the indicator weights. As one of many multiple criteria decision-making methods, ANP is frequently used to determine the weights of indicators. ANP is basically an extension of the well-known Analytic Hierarchy Process (AHP). The obvious advantage of ANP over AHP is that it can handle the interdependence and feedback among or within groups of criteria in a network structure model [40]. The typical ANP model includes alternatives, criteria, and networks of influence with dependencies and feedbacks. Each decision network consists of clusters, elements, and the links between elements. However, in complex decision-making problems, it is assumed by default that the same weight of each cluster can cause problems of neglecting the different degrees of dependence or feedback among criteria [41]. To solve this problem, the DEMATEL method can be adopted [42]. DEMATEL can effectively construct a network relation map with clear interrelations among or within each criterion. Besides, the relative weights of criteria are considered as reciprocal values in the traditional ANP. To eliminate this weakness, DEMATEL can be adopted and the method is more advantageous for satisfying the actual system where independences and feedbacks between groups of elements are determined more objectively based on a network relation map [41]. In recent literature, the method of integrating DEMATEL with ANP has already been used to determine the degree of mutual influence among criteria and to normalize the unweighted super matrix [43]. Therefore, by combining the characteristics of the methods and the CUPS resilience indicator system, a hybrid approach integrating DEMATEL with ANP was adopted to determine the weights of indicators.

#### 4.2.1. DEMATEL

DEMATEL has been developed and widely accepted as one of the best tools to present the cause and effect relationships between assessment criteria [44,45]. The interdependencies among criteria are demonstrated through a network relation map (NRM). Besides, the method is also used to obtain the level of impact of each element relative to other elements.

Step 1. Calculate the initial average matrix scores. m experts and n factors are determined. Each expert is invited to judge the degree of direct influence between two elements based on a pairwise comparison. The comparison scale has five levels: no influence (0), low influence (1), medium influence (2), high influence (3), and very high influence (4) [45]. The m direct influence matrices can be generated by experts’ judgment. Subsequently, the initial average matrix can be calculated by the same element of the m direct influence matrices that is an n×n matrix A, in which $a_{ij}$ is the degree that the element i affects the element j.

Step 2. Calculate the initial influence matrix. The normalized initial influence matrix D is derived from the normalized average matrix A through Equation (9).

(9) $$D=s\times A,\;s=\min\left[\frac{1}{\underset{i}{\max}\sum_{j=1}^{n}a_{ij}},\frac{1}{\underset{j}{\max}\sum_{i=1}^{n}a_{ij}}\right]$$

Step 3. Derive the total-influence matrix T. As the power of $D(\underset{k\to \infty }{\lim}\left(D=,{\left[0\right]}_{n\times n}\right)$ increases, the indirect effects between the indicators decrease continuously. The total-influence matrix T can be calculated using Equation (10), indicating the total influence obtained from the combination of indirect influences among all elements. I is an n×n identity matrix.

(10) $$T{=D+D}^{2}+\cdots {+D}^{\infty }=D{\left(I-D\right)}^{-1}$$

Furthermore, $R_{i}$ and $C_{j}$ are defined as the sum of elements $t_{ij}$ of the matrix T by rows and columns:

(11) $${R=(R}_{1},R_{2},\ldots ,R_{n}),\;R_{i}=\sum_{j=1}^{n}t_{ij},\;(i=1,2,\ldots ,n)$$

(12) $$C={\left(C_{1},C_{2},\ldots ,C_{n}\right)}^{T},\;C_{j}=\sum_{i=1}^{n}t_{ij},\;(j=1,2,\ldots ,n)$$

where $R_{i}$ denotes the sum of the direct and indirect effects of the i th row element relative to other elements. Similarly, $C_{j}$ is the sum of the direct and indirect effects of the j th column element received from other elements. Accordingly, when j=i, the value of $\left(R_{i}+C_{j}\right)$ represents the magnitude of the intensity of influences given and received, which shows that element i plays a central role in the problem. In contrast, the value of $\left(R_{i}-C_{j}\right)$ represents the net contribution of element i to the system. In addition, if $\left(R_{i}-C_{j}\right)$ is positive, it means that element i affects other elements and is called the dispatcher. If $\left(R_{i}-C_{j}\right)$ is negative, it indicates that element i is affected by other elements and is called the receiver.

Step 4. Set a threshold value and obtain the NRM. In practice, if all the information from the matrix T is converted to an NRM, the map is too complex to show the necessary information. Therefore, the threshold value α should be determined, which is calculated as the average of the elements $t_{ij}(i,j=1,2,\ldots ,n)$ of the matrix T. The determination of the threshold value offers a basis for filtering out the negligible effects in the elements of matrix T. If “$t_{ij}>\alpha$”, it indicates that the influence value is high and can be converted into the NRM. When the indicators of the relative high influence value are determined, the network relation map can be drawn [46].

#### 4.2.2. ANP

After constructing the network relation map, the weights of indicators are calculated by using ANP. ANP considers various forms of dependence and feedback. When calculating the weighted super-matrix, each cluster is considered to be the same weight by default. It neglects the fact that the degree of mutual influence among criteria may be different [41,47]. To overcome the mentioned drawbacks, the total-influence matrix is applied to calculate the weight of the indicator. The combination of DEMATEL and ANP (DANP) is much closer to real circumstances. The cause and effect relationships of criteria can be obtained through the above four steps of DEMATEL, and ANP can then confirm the influence degree of criterion. The DANP method is presented as the following steps [47,48].

Step 5. Determine the unweighted super-matrix. Before determining the unweighted super-matrix, it is necessary to construct the network model by the ANP method based on the total-relation matrix $T_{c}$. Firstly, the total-influence matrix T (Equation (13)) should be normalized.

(13) $$T_{c}=T=\left[T_{c}^{ij}\right],i,j=1,2,\ldots ,n$$

where the matrix $T_{c}^{ij}$ contains the factors from cluster $D_{i}$ and influences other factors from cluster $D_{j}$.

Step 5.1. Normalize the total-relation matrix $T_{c}$. During the process of normalization, $T_{c}^{\alpha }$ (Equation (14)) can be obtained by $T_{c}$. For instance, the normalization process of $T_{c}^{\alpha 11}$ is shown as Equation (15).

(14) $$T_{c}^{\alpha }=\left[T_{c}^{\alpha \mathrm{ij}}\right],i,j=1,2,\ldots ,n$$

(15) $$T_{c}^{\alpha 11}=\left[t_{c^{ij}}^{\alpha 11}\right]=\left[t_{c^{ij}}^{11}/d_{c^{i}}^{11}\right],\;d_{c^{i}}^{11}=\sum_{j=1}^{m_{1}}t_{ij}^{11},\;i,j=1,2,\ldots m_{1}$$

where $t_{c^{ij}}^{11}$ is the factor influence value from cluster $D_{1}$ and element $t_{c^{ij}}^{\alpha 11}$ is the normalized value.

Step 5.2. Calculate the unweighted super-matrix. The super-matrix $W^{ij}$ of each cluster can be obtained by transposing the relevant clusters of the normalized total-influence matrix $T_{c}^{\alpha }$. The unweighted super-matrix W is shown as Equation (16). For example, the matrix $W^{11}$ is a collection of factor influence values connected with factors in cluster $D_{1}$.

(16) $$W={{(T}_{c}^{\alpha })}^{T}=\left[W^{\mathrm{ij}}\right],\;i,j=1,2,\ldots ,n$$

(17) $$W^{11}=\left[t_{c^{ij}}^{\alpha 11}\right],\;i,j=1,2,\ldots ,m_{1}$$

Step 6. Obtain the weighted super-matrix. The elements of the weighted super-matrix $W^{\alpha }$ can be calculated by multiplying the elements of the unweighted super-matrix W by the corresponding elements obtained from the normalized total influence matrix $T_{D}^{\alpha }$ (Equation (18)). Then, the elements of the weighted super-matrix are obtained by normalizing the total-influence matrix, as shown in Equations (19) and (20).

(18) $$T_{D}^{\alpha }=\left[t_{D}^{\alpha ij}\right]=\left[t_{D}^{ij}/d_{i}\right]\;,\;d_{i}=\sum_{j=1}^{n}t_{D}^{ij},\;i,j=1,2,\ldots ,n\;$$

where $d_{i}$ is the sum of values of the cluster $D_{i}$ from the total influence-matrix $T_{D}$. The total-influence matrix $T_{D}$ (Equation (19)) can be calculated by the initial total-influence matrix T. In particular, $t_{D}^{11}$ is the average of the values of factors in cluster $D_{1}$ from matrix T.

(19) $$T_{D}=\left[t_{D}^{ij}\right],i,j=1,2,\ldots n,\;t_{D}^{11}=\frac{1}{n\times m}\sum_{i=1}^{n}\sum_{j=1}^{m}t_{ij}^{11}$$

(20) $$W^{\alpha }={\left(T_{D}^{\alpha }\right)}^{T}*W=\left[t_{D}^{\alpha ij}\times W^{ij}\right],\;i,j=1,2,\ldots ,n$$

Step 7. Limit the weighted super-matrix. The weighted super-matrix $W^{\alpha }$ can be multiplied by itself multiple times to obtain a limited super-matrix, thereby obtaining the weight of each cluster. Afterwards, the weighted super-matrix can be raised to the limiting powers until the super-matrix has converged and become a long-term stable super-matrix to obtain the global weight, which is called the DANP weight, such as $\underset{k\to \infty }{\lim}{{(W}^{\alpha })}^{k}$.

## 5. Results

To validate the proposed methodology, case studies for the Chongqing West Railway Station (the Station (W)) and Lianglukou Rail Transit Station (the Station (L)) were conducted.

### 5.1. The Chongqing West Railway Station and Lianglukou Rail Transit Station

The Chongqing West Railway Station is the largest integrated passenger transportation hub in western China. The station has five floors, covering an area of 127,000 square meters [49]. According to the Chongqing Medium- and Long-Term Railway Network Planning (2016–2030), the capability of arrival and departure passengers of the station is designed to be 42.18 million, with approximately 381 pairs of trains per day [50]. Since its operation on 25 January 2018, there have been about 59 pairs of trains per day, with about 70,000 passengers.

The Lianglukou Rail Transit Station is located at Yuzhong District, Chongqing, China. The station is the transfer station of Monorail Line 1 and Line 3. Four floors make up the three-dimensional space of the station and are connected through stairs or escalators. Since its operation on 28 July 2011, the transfer volume of the station has been the largest every year in Chongqing. According to the annual transportation development reports for the past seven years, the average daily transfer passenger volume has increased from 73,300 to 191,000.

### 5.2. Data Collection

The process of data collection can be divided into three phases. Firstly, different considerations and decisions related to the assessment objectives and criteria were discussed. Then, three field investigations for each of the case stations were conducted and information for determining the scores of indicators in level 3 was collected. The score of preparedness or vulnerability was divided into five levels: low (1), relatively low (2), general (3), high (4), and very high (5). Based on the field investigation results, the scores of indicators in level 3 were obtained. Finally, a pair-wise comparison was conducted between every two indicators in the two indicator systems, respectively. As a result, the direct influence matrices from the DEMATEL method were generated.

### 5.3. Evaluation Results

#### 5.3.1. Score Determination of Indicators in Level 3

As shown in Table 2 and Table 3, indicators in level 3 can be divided into two categories: qualitative indicators and quantitative indicators. The scores of qualitative indicators were obtained by judging the actual situation of every indicator, while the scores of quantitative indicators were calculated by the data from field investigations. Some indicators are difficult to compare directly due to the different magnitude of the two cases, so 100 square meters were used as the comparison unit. For instance, the amount of fire-fighting equipment per 100 square meters at Station (W) was 0.312, and that of Station (L) was 0.451. Therefore, the corresponding indicator score was 3 and 4.

#### 5.3.2. Weight Calculation of Indicators in Level 2

**Figure 2:**
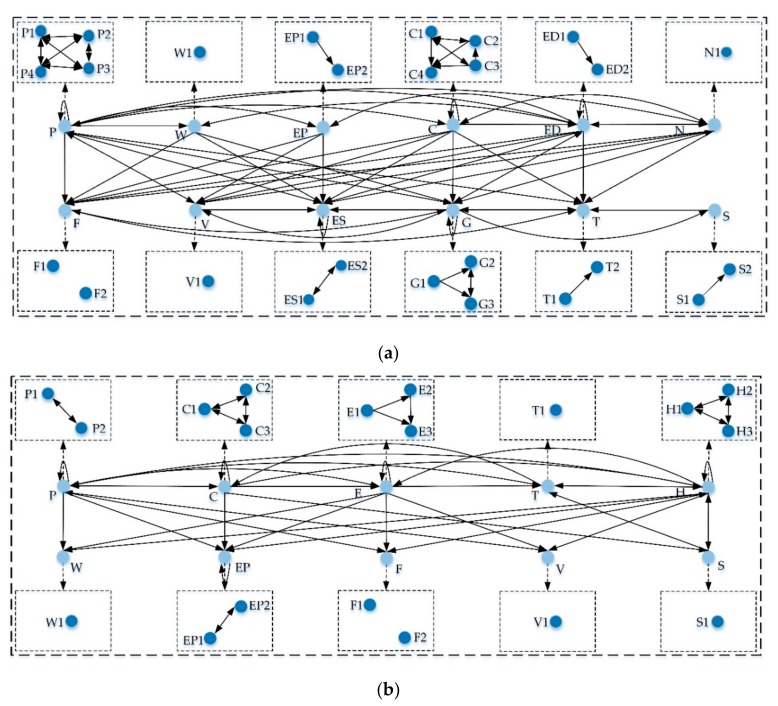
Network relation map; (**a**) Preparedness; (**b**) vulnerability.

Firstly, the threshold values of indicators in level 2 and level 1 were obtained by calculating the average of the elements in the matrices $T_{c}$ and $T_{D}$, respectively. The network relation maps of preparedness and vulnerability are shown in Figure 2. As illustrated in Figure 2a, the evaluation results were divided into a dispatcher group, including *P*, *W*, *EP*, *C*, *ED*, and *N*, and a receiver group consisting of *F*, *V*, *ES*, *G*, *T*, and *S*. Figure 2a shows the central role of *physical structure* in disaster preparedness. As for the vulnerability, Figure 2b illustrates the network relationships between indicators in level 1 and level 2. The dispatcher group’s indicators include *P*, *C*, *E*, *T*, and *H*, while the receiver group consists of *W*, *EP*, *F*, *V*, and *S*. Indicator *H* (*hazard*) influenced all indicators except indicator *P* (*physical structure*), with the three indicators in level 2 interacting with each other.

Through steps 5 and 6, the weighted super-matrix can be obtained. Subsequently, the final limited super-matrix can be established by calculating the limiting power of the weighted super-matrix until the stable state was reached. The limited super-matrix was obtained through a series of data processing. The final limited super-matrix can be calculated by averaging the corresponding elements’ values of the N super-matrices [41]. In addition, the super-matrix can be selected provided that the sum of the element values in the super-matrix equals 1. Therefore, under the premise that the sum of all element values in the super-matrix retained one decimal place, the final limited super-matrix of the preparedness was determined by three super-matrices, and the vulnerability was calculated by two super-matrices. The global weights of indicators for the preparedness and vulnerability are presented in Table 2 and Table 3.

#### 5.3.3. Resilience Evaluation Score

The evaluation results of the preparedness and vulnerability were calculated by using Equations (7) and (8). For Station (W), the scores of preparedness and vulnerability were 0.3176 and 0.2453, respectively. Therefore, the resilience score was 1.2950, indicating that the station was more resilient to risks. The disaster preparedness is adequate to cope with its vulnerability and can effectively respond to disasters. For Station (L), the scores of preparedness and vulnerability were 0.2906 and 0.3566, respectively. Therefore, the resilience score was 0.8149, indicating that the station was less resilient to risks.

## 6. Results Analysis 

### 6.1. Results Analysis of “Preparedness”

The preparedness score of Station (W) is 0.3176 and higher than that of Station (L). As shown in Table 2, for Station (W), the last four indicators with lower scores are *W*, *N*, *S*, and *V*. For Station (L), the scores of *W*, *V*, *S*, and *C* are relatively low. In terms of indicators *W*, *S*, and *V*, their weights are very low (0.048, 0.049, and 0.072, respectively). Figure 2a shows that indicator *W* (dispatcher) has no effect on the other two indicators *S* and *V* (receivers). It demonstrates that the indicators have little impact on system disaster preparedness. For indicator *W*, the two stations have the same scores, showing that the preparation of *water supply and drainage facilities* can satisfy normal operations and effectively cope with emergencies. For indicator *S*, the score of Station (W) is slightly higher than that of Station (L). The result meets the reality that more emergency volunteers and NGOs in Station (W) are arranged to maintain normal operations and respond to emergencies than in Station (L). The score of *V* of Station (W) is higher than that of Station (L). According to the field investigation, the air environment of Station (W) is better than that of Station (L). Station (L) is located in an underground space, and the environment is a little stuffy during the peak period.

Additionally, the scores of the indicators *N* and *C* of the two stations are different and the two indicators are dispatchers. Figure 2a shows that indicators *N* and *C* affect indicator *V*, and there are interactions among the four sub-levels of indicator *C*. Firstly, considering indicator *N*, the possibility of natural disasters is at a low level according to the historical statistics for both stations. Then, for Station (L), although the security protection for crowd clusters is highly valued, the passengers’ congestion phenomenon is still serious, which is reflected by the scores of *C1*, *C2*, and *C3*. As the number of passenger flows in the spatial and temporal distributions increases, the statistics of passenger flows will increase significantly. This fully reflects the interaction among the above three indicators and satisfies the reality. Moreover, passenger congestion causes a poor air quality in Station (L), which is reflected by the low score of *V*. In addition, according to the field investigation, the *automatic alarm systems* in Station (L) have a higher density than those of Station (W), which is also reflected by the low score of *F2* of Station (W). Besides, traffic congestion often occurred around Station (W). There is an urgent need to alleviate these problems. Based on the above analysis, there are general and individual problems in the operations of the two stations. Therefore, measures should be taken to improve the different situations of both stations from the above two perspectives.

In response to the individual problems, two suggestions are made for Station (W): (1) Increase the number of automatic alarm systems for emergencies and (2) Optimize roads to alleviate traffic congestion around Station (W). For Station (L), the following three suggestions are made: (1) Increase the number of volunteers or NGOs to provide transportation guidance for passengers; (2) check the ventilation system regularly and maintain a comfortable environment; (3) and control passenger flow and increase subway lines to alleviate congestion and reduce risks. For the general problems, two suggestions are made for the sustainable development of the two stations: (1) Strengthen daily coordination among multi-stakeholders to maintain continuous and stable operations and (2) collect passengers’ opinions extensively to meet diverse needs and achieve quality services.

### 6.2. Results Analysis of “Vulnerability”

The vulnerability score of Station (W) and Station (L) is 0.2453 and 0.3566, respectively. As shown in Table 3, for Station (W), four indicators with higher scores were *P*, *H*, *C*, and *F*. For Station (L), the scores of *P*, *H*, *C*, and *V* were relatively higher throughout the indicator system.

In terms of indicators *P*, *H*, and *C*, the weights are 0.131, 0.174, and 0.090, respectively. Figure 2b shows that all three indicators are dispatchers which interact with each other. This indicates that they have great impacts on the system vulnerability. Firstly, regarding indicator *P*, the score of Station (W) was lower than that of Station (L), which was mainly attributed to the indicator *P2*. The result implies that the *internal spatial layout* of Station (W) could be relatively conducive for passenger flow transfer and passenger congestion alleviation. Specifically, Station (W) has more emergency passages than Station (L). The weight of indicator *H* is the largest, indicating that system vulnerability can be mainly attributed to *hazard*. Potential risks are generally hidden in vulnerable points, such as escalators, elevators, and security gates, with crowded clusters. Therefore, the vulnerability score of Station (L) was higher than that of Station (W). For Station (W), both scores of *H1* and *H2* are higher than that of *H3*. This meets the reality that the incidence of hazard in Station (W) was relatively low during the first operation year. For Station (L), the scores of sub-indicators are the same (2.5), indicating that a rich emergency experience and sufficient disaster preparedness can permit an effective response to risk events and quickly resume a normal operation. The indicator *C* reflects the distribution of crowd clusters in the CUPSs. For Station (W), both of the scores of *C2* and *C3* are more than 3, which means that the distribution of crowd clusters in Station (W) is exposed to potential risks. Crowd clusters are mainly concentrated in Station (L), so three indicators in level 2 have high scores.

Moreover, the scores of the indicators *F* and *V* of the two stations are different and the two indicators are receivers. For Station (W), the score of *F* is relatively high, and Figure 2b shows the interaction of each of the two indicators among indicators *P*, *H*, and *F*. It indicates that *fire protection facilities* play an important role in avoiding risk and reducing vulnerability. According to the field investigation, the number of *fire protection facilities* on the concourse floor is relatively small. In general, fires are caused by human factors and equipment accidents. The incidence of fires and explosion events increases as the number of passengers increases. Therefore, investment in the fire protection facilities in Station (W) should be increased. For Station (L), the score of *V* is high, and Figure 2b shows that the physical structure and occurrence of hazards also have impacts on the ventilation system of the station.

Considering the different situations of both stations, different measures are proposed. The following two suggestions are made for Station (W): (1) Arrange more security policemen and prepare relief supplies to respond to emergencies and (2) inspect and replace fire protection facilities regularly to deal with fires and explosions. For Station (L), the following two suggestions are made: (1) Arrange security policemen to ease passenger congestion and (2) strengthen disaster prevention propaganda and conduct emergency drills regularly to improve passengers’ awareness of safety protection. For the general problems, increasing financial investment to improve facilities and the emergency reserves is necessary for systemic vulnerability reduction and potential risk migration.

## 7. Conclusions

CUPSs are frequently affected by disasters due to their complex structure and crowded clusters. This paper has formulated a theoretical framework to assess the resilience of CUPSs. According to the implication of resilience, it is defined as the ratio of disaster preparedness to system vulnerability. Three-level indicator systems were established for the two dimensions, respectively. A hybrid approach was introduced in the resilience assessment that combined the Analytic Network Process (ANP) and the Decision-Making Trial and Evaluation Laboratory (DEMATEL). Furthermore, case studies on the Chongqing West Railway Station and Lianglukou Rail Transit Station were conducted to validate the proposed methodology. The methodology could be improved according to the different characteristics of CUPSs. The resilience assessment of the CUPSs is conducive to improving the design and operation of urban infrastructures. The assessment results could provide useful references for urban managers and decision makers.

This research is a first step in developing the methodology to assess the resilience of CUPSs. The system resilience value could be calculated through the proposed methodology. However, the sensitivity of the system resilience value for the variation of different indicators could not be determined. Strategies that improve the system resilience level also could not be established pertinently. Therefore, the resilience simulation of CUPSs is a further direction for future research. In the simulation, the variation of system resilience could be simulated based on the variation of different indicators. Therefore, the important indicators for the system resilience could be determined, and useful strategies could then be formulated to improve the system resilience.

## Figures and Tables

**Table 1:** Resilience assessment indicators.

References	Objects	Indicator Categories
Cutter et al. [21]	community	social, economic, institutional, infrastructure, ecological, community competence
Cutter et al. [22]	community	social, economic, institutional, infrastructure, community capital
Sherrieb et al. [23]	community	social capital, economic development
Frazier et al. [24]	community	social, economic, institutional, infrastructure, community capital, regulatory, ecological, temporal, spatial
Pfefferbaum et al. [25]	community	connection and caring, resources, transformative potential, disaster management, information and communication
Cutter et al. [26]	community	social, economic, institutional, infrastructure, community capital, environmental
Kusumastuti et al. [9]	community	social, economic, institutional, infrastructure, community capacity, hazard
Burton [27]	community	social, economic, institutional, infrastructure, community capital, environmental systems
Cimellaro et al. [28]	community	social-cultural capital, economic development, organized governmental services, physical infrastructures, population and demographics, environmental and ecosystem, lifestyle and community competence
Suárez et al. [29]	urban system	self-sufficiency, business diversity, land use diversity, food diversity, spaces for citizen participation
Xu and Xue [1]	complex urban public spaces	economy development, government governance, physical structure, crowd cluster, natural environment, traffic accessibility,
Wang et al. [30]	infrastructure	demand, status, influence, resource, measure
Sharifi and Yamagata [31]	urban system	society and well-being, economy, governance and institution, built environment and infrastructure, materials and environmental resources

**Table 2:** The indicator system of “preparedness”.

Level 1	Weight	Score	Score	Level 2	Weight	Score	Score	Level 3	Score	Score
		(W)	(L)			(W)	(L)		(W)	(L)
Physical structure (P)	0.098	0.290	0.241	P1: Multi-layer structure	0.011	1.750	2.250	P1.1: The number of layers of CUPSs	2	1
								P1.2: The height of the atrium	2	4
								P1.3: The number of layers of underground spaces	1	2
								P1.4: The height of each layer of CUPSs	2	2
				P2: Internal spatial layout	0.035	3.500	2.833	P2.1: The total number of escalators	2	4
								P2.2: The total number of elevators	4	2
								P2.3: The total number of emergency passages	4	2
								P2.4: The number of security checkpoints	4	2
								P2.5: The number of entrances and exits	4	4
								P2.6: The number of columns in the space	3	3
				P3: Underground spatial layout	0.032	4.000	2.000	P3.1: The coverage of the pedestrian passage	4	2
								P3.2: The coverage of the business zone	4	2
				P4: Multi-function	0.020	1.000	2.667	P4.1: The number of the function	1	3
								P4.2: The category of the function	1	3
								P4.3: The connection of the function	1	2
Water supply and drainage preparation (W)	0.048	0.144	0.144	W1: Water supply and drainage facilities	0.048	3.000	3.000	W1.1: The number of emergency water supply facilities	3	3
								W1.2: The number of emergency water drainage facilities	3	3
Electronic power system (EP)	0.058	0.232	0.193	EP1: Emergency power supply equipment	0.039	4.000	3.000	EP1.1: The category of emergency power supply equipment	4	3
								EP1.2: The amount of emergency power supply equipment	4	3
				EP2: Power condition	0.019	4.000	4.000	EP2.1: Voltage qualification rate	4	4
								EP2.2: Emergency lighting distribution rate	4	4
Fire protection facilities (F)	0.106	0.285	0.387	F1: Fire-fighting equipment	0.055	3.333	3.333	F1.1: The amount of fire-fighting equipment	3	4
								F1.2: The category of fire-fighting equipment	4	2
								F1.3: The amount of fire-fighting equipment in each layer of the CUPSs	3	4
				F2: Automatic alarm system	0.051	2.000	4.000	F2.1: The number of automatic fire alarm systems	2	4
								F2.2: The number of automatic fire alarm systems in each layer of the CUPSs	2	4
Ventilation system (V)	0.072	0.216	0.144	V1: Ventilation facilities and equipment	0.072	3.000	2.000	V1.1: The number of air shafts	3	2
								V1.2: Installation of the air conditioning system	3	2
Environmental sanitation (ES)	0.107	0.365	0.241	ES1: Indoor environment of the CUPSs	0.060	3.333	1.667	ES1.1: Indoor temperature and humidity	4	2
								ES1.2: Indoor volume decibel	3	1
								ES1.3: The cleanliness of the indoor environment	3	2
				ES2: Health protection measures	0.047	3.500	3.000	ES2.1: The number of cleaners in the CUPSs	3	2
								ES2.2: The number of sanitation and epidemic prevention personnel	4	4
Characteristics of passenger flows (C)	0.066	0.239	0.156	C1: Statistics of passenger flows	0.010	3.000	1.333	C1.1: The designed capacity of the CUPSs	2	2
								C1.2: The average daily volume of inbound passenger flows	4	1
								C1.3: The average daily volume of outbound passenger flows	3	1
				C2: Spatial distribution of passenger flows	0.014	2.000	1.500	C2.1: The maximum density of passenger volume	1	1
								C2.2: The distribution of passenger flows in each layer of the CUPSs	3	2
				C3: Temporal distribution of passenger flows	0.004	2.500	2.000	C3.1: Daily distribution of passenger flows	3	1
								C3.2: Annual distribution of passenger flows	2	3
				C4: Security protection for crowd clusters	0.038	4.500	3.000	C4.1: The average number of policemen for every crowd cluster	5	3
								C4.2: The amount of equipment and number of facilities for every crowd cluster	4	3
Government governance (G)	0.151	0.574	0.489	G1: Multi-stakeholder cooperation	0.017	3.500	4.000	G1.1: The number of governance departments in the CUPSs	4	4
								G1.2: Closeness of cooperation among different departments	3	4
								G1.3: The time period of multi-stakeholder cooperation	3	4
								G1.4: The degree of effectiveness of multi-stakeholder cooperation	4	4
				G2: Disaster emergency plan	0.065	3.667	3.500	G2.1: The number of overall emergency plans	4	4
								G2.2: The number of special emergency plans	3	3
								G2.3: The frequency of emergency drills	4	3
								G2.4: The number of professional rescuers	4	4
								G2.5: Professional rescuers’ training	3	3
								G2.6: The number of rescue material reserves	4	4
				G3: Disaster management plan	0.069	4.000	2.800	G3.1: The number of risk assessment measures	4	3
								G3.2: The number of risk mitigation plans	3	3
								G3.3: Strength of propaganda on risk event prevention	4	3
								G3.4: The number of evacuation signs in the CUPSs	4	3
								G3.5: The number of evacuation signs in each layer of the CUPSs	4	3
								G3.6: The number of emergency escape routes	4	2
								G3.7: The number of security policemen	5	2
								G3.8: The category and number of security facilities and equipment	5	2
								G3.9: The proportion of floating population exposed to disaster management education	4	4
								G3.10: The number of disaster management education activities per year	3	3
Economic development (ED)	0.096	0.358	0.369	ED1: Economic support	0.044	4.000	4.250	ED1.1: Regional gross domestic product	3	5
								ED1.2: Financial allocation to the CUPSs	5	4
								ED1.3: Financial reserves for the emergency rescue after risk events	4	4
								ED1.4: Financial reserves for the post-disaster reconstruction	4	4
				ED2: Prepared rescue materials	0.052	3.500	3.500	ED2.1: The number of prepared rescue materials	4	4
								ED2.2: The degree and category that the rescue materials could respond to	3	3
Traffic accessibility (T)	0.117	0.257	0.309	T1: Traffic condition	0.019	1.500	2.500	T1.1: The number of connected roads	2	3
								T1.2: Traffic flow operation status	1	2
				T2: Emergency rescue	0.098	2.333	2.667	T2.1: The unimpeded nature of special emergency routes	2	2
								T2.2: Emergency rescue vehicles’ accessibility	2	3
								T2.3: Rescuers’ accessibility	3	3
Social cooperation (S)	0.049	0.172	0.147	S1: Social preparedness	0.024	3.000	3.000	S1.1: The proportion of floating population with emergency vehicles	3	3
								S1.2: The proportion of floating population covered by medical services	3	3
				S2: Social service	0.025	4.000	3.000	S2.1: The number of emergency volunteers	4	3
								S2.2: The number of emergency nongovernmental organizations	4	3
Natural environment (N)	0.054	0.162	0.162	N1: The statistics of multi-category natural disaster	0.054	3.000	3.000	N1.1: The statistics of strength for natural disasters	3	3
								N1.2: The statistics of incidence for natural disasters	3	3

**Table 3:** The indicator system of “vulnerability”.

Level 1	Weight	Score	Score	Level 2	Weight	Score	Score	Level 3	Score	Score
		(W)	(L)			(W)	(L)		(W)	(L)
Physical structure (P)	0.131	0.368	0.580	P1: Multi-layer structure	0.020	4.500	4.000	P1.1: The complexity of topography in the CUPSs	5	4
								P1.2: The number of layers of the CUPSs	4	4
				P2: Internal spatial layout	0.111	2.500	4.500	P2.1: The load of escalators	2	5
								P2.2: The load of elevators	3	4
								P2.3: The number of emergency passage distributions	1	4
								P2.4: The volume of passenger flow at security checkpoints	4	5
Water supply and drainage preparation (W)	0.080	0.120	0.160	W1: Water supply and drainage facilities	0.080	1.500	2.000	W1.1: The failure rate of the emergency water supply facilities	2	2
								W1.2: The failure rate of the emergency water drainage facilities	1	2
Electronic power system (EP)	0.124	0.141	0.248	EP1: Emergency power supply equipment	0.073	1.000	2.000	EP1.1: The failure rate of the emergency power supply equipment	1	2
				EP2: Power condition	0.051	1.333	2.000	EP2.1: Voltage disqualification rate	2	2
								EP2.2: The frequency of average interruption	1	2
								EP2.3: The average interruption duration	1	2
Fire protection facilities (F)	0.103	0.235	0.384	F1: Fire-fighting equipment	0.057	2.500	3.500	F1.1: The obsolete rate of fire-fighting equipment	3	4
								F1.2: The failure rate of fire-fighting equipment	2	3
				F2: Automatic alarm system	0.046	2.000	4.000	F2.1: The failure rate of the automatic alarm systems	2	4
Ventilation system (V)	0.097	0.194	0.388	V1: Ventilation facilities and equipment	0.097	2.000	4.000	V1.1: The failure rate of the air conditioning system	2	4
Characteristics of internal vulnerability (C)	0.090	0.262	0.404	C1: Crowd cluster statistics	0.032	2.000	4.000	C1.1: The frequency of passenger traffic overload	2	4
				C2: Spatial distribution of crowd cluster	0.030	3.333	5.000	C2.1: The number of crowd clusters	3	5
								C2.2: The location of crowd clusters	3	5
								C2.3: The crowd cluster density of population in each layer	4	5
				C3: Temporal distribution of crowd cluster	0.028	3.500	4.500	C3.1: Daily distribution of crowd clusters	3	5
								C3.2: Annual distribution of crowd clusters	4	4
Economy (E)	0.084	0.200	0.241	E1: Regional economic condition	0.016	4.000	3.000	E1.1: Regional average expense	4	3
								E1.2: Portion of regional expense for daily needs	4	3
				E2: Financial reserves	0.023	2.000	2.500	E2.1: The financial reserves for post-disaster emergency rescue	2	2
								E2.2: The financial reserves for post-disaster reconstruction	2	3
				E3: Rescue material reserves	0.045	2.000	3.000	E3.1: The reserves for post-disaster rescue material	2	3
Traffic (T)	0.068	0.204	0.238	T1: Traffic condition	0.068	3.000	3.500	T1.1: Traffic accessibility	4	3
								T1.2: Public transportation service capacity	2	4
Social cooperation (S)	0.062	0.186	0.186	S1: Social circumstance	0.062	3.000	3.000	S1.1: The coverage of emergency vehicles	3	3
								S1.2: The coverage of medical services	3	3
Hazard (H)	0.174	0.352	0.435	H1: Variety	0.037	2.750	2.500	H1.1: The number of natural disasters	2	1
								H1.2: The number of accident disasters	2	3
								H1.3: The number of public health accidents	3	2
								H1.4: The number of social security accidents	4	4
				H2: Characteristics	0.045	2.500	2.500	H2.1: The strength of emergencies	3	2
								H2.2: The frequency of emergencies	2	3
				H3: Severity	0.092	1.500	2.500	H3.1: The degree of loss in the CUPSs	1	3
								H3.2: Disaster-prone areas of the CUPSs	2	4
								H3.3: The number of injured casualties in the CUPSs’ disaster area	1	1
								H3.4: The loss caused by disasters per year	2	2
